# Caspofungin as antifungal prophylaxis in pediatric patients undergoing allogeneic hematopoietic stem cell transplantation: a retrospective analysis

**DOI:** 10.1186/1471-2334-12-151

**Published:** 2012-07-02

**Authors:** Michaela Döring, Ulrike Hartmann, Annika Erbacher, Peter Lang, Rupert Handgretinger, Ingo Müller

**Affiliations:** 1 Department of Pediatric Hematology/Oncology, University Children’s Hospital Tübingen, Hoppe-Seyler-Str.1, 72076, Tübingen, Germany; 2 University Pharmacy, University Children’s Hospital Tübingen, Hoppe-Seyler-Str.1, 72076, Tübingen, Germany; 3 Present address: Clinic of Pediatric Hematology and Oncology, University Medical Center Hamburg-Eppendorf, Martinistr. 52, 20246, Hamburg, Germany

**Keywords:** Caspofungin, Liposomal amphotericin B, Antifungal prophylaxis, Pediatric patients, Allogeneic stem cell transplantation

## Abstract

**Background:**

Pediatric patients undergoing allogeneic hematopoietic stem cell transplantation (HSCT) often receive intravenous liposomal amphotericin B (L-AmB) as antifungal prophylaxis. There are no guidelines for antifungal prophylaxis in children in this situation. Caspofungin (CAS), a broad-spectrum echinocandin, could be an effective alternative with lower nephrotoxicity than L-AmB.

**Methods:**

We retrospectively analyzed the safety, feasibility, and efficacy of CAS in our center, and compared the results with L-AmB as antifungal monoprophylaxis in pediatric patients undergoing HSCT. 60 pediatric patients received L-AmB (1 or 3 mg/kg bw/day) and another 60 patients received CAS (50 mg/m^2^/day) as antifungal monoprophylaxis starting on day one after HSCT. The median ages of patients receiving L-AmB and CAS were 7.5 years and 9.5 years, respectively.

**Results:**

No proven breakthrough fungal infection occurred in either group during the median treatment period of 23 days in the L-AmB group and 24 days in the CAS group. One patient receiving CAS developed probable invasive aspergillosis. During L-AmB treatment, potassium levels significantly decreased below normal values. Patients treated with L-AmB had more drug-related side effects and an increased need for oral supplementation with potassium, sodium bicarbonate and calcium upon discharge as compared with the CAS group. CAS was well-tolerated and safe in this cohort of immunocompromised pediatric patients, who underwent high-dose chemotherapy and HSCT.

**Conclusion:**

Prophylactic CAS and L-AmB showed similar efficacy in this biggest cohort of pediatric patients after allogeneic HSCT reported, so far. A prospective randomized trial in children is warranted to allow for standardized guidelines.

## Background

Pediatric patients undergoing hematopoietic stem cell transplantation (HSCT) are at high risk of acquiring severe invasive opportunistic fungal infections. Risk factors include extensive immunosuppression, cytopenia, T-cell depletion of the graft, graft-versus-host disease (GvHD), underlying disease and viral or bacterial infections [1,2]. *Candida* and *Aspergillus* species are the most common fungal pathogens in this setting, with *Aspergillus* infections being associated with higher mortality [3-6]. Studies on intravenous antifungal prophylaxis regimens in pediatric patients undergoing HSCT are scarce and included 51 subjects or less. Hence, a variety of strategies for the prophylaxis, empiric, pre-emptive and targeted treatment of invasive fungal infections have been published [7-10].

Pediatric patients (< 18 years) most frequently received antifungal prophylaxis with liposomal amphotericin B (L-AmB) after allogeneic HSCT [10-12]. However, nephrotoxicity or infusion-related side effects have been reported in some studies in adolescents and adults [13-15]. In a randomized, multicenter trial with 343 neutropenic pediatric and adult patients empirically treated with L-AmB, side effects included creatinine increase (19% of cases), fever (17%), and rigor (18%) [16]. Maculopapular rash, itching, and hyperphosphatemia were reported in case reports of pediatric patients when L-AmB was used [17,18].

Caspofungin (CAS) was shown to be effective in treatment of invasive candidiasis and aspergillosis with low toxicities in adults [19-21]. In children, CAS proved to be effective in the primary treatment of candidiasis [22]. In retrospective studies of immunocompromised pediatric patients, the safety and tolerability of CAS was favorable [23,24]. The tolerability, safety, and efficacy of CAS and L-AmB have also been compared in the empirical antifungal treatment of febrile neutropenic pediatric patients [25]. Based on these experiences, CAS was considered for antifungal prophylaxis with potentially fewer nephrotoxic side effects than L-AmB and employed in pediatric patients after allogeneic HSCT. Retrospectively, we analyzed safety and efficiency of CAS and L-AmB.

## Methods

This retrospective survey was conducted in accordance with the declaration of Helsinki and performed with approval by the University Children’s Hospital Tübingen’s Institutional Review Board. Data were collected retrospectively, entered in a standardized case report form, and anonymized.

### Study design

The study is a single centre, retrospective survey on antimycotic prophylaxis in pediatric patients under eighteen years of age who underwent allogeneic HSCT between January 2006 and June 2010 at the University Children’s Hospital Tübingen, Germany. The allogeneic stem cell transplantation occurred in high-efficiency particle-arrestance filtered environment. All pediatric patients in our clinic received L-AmB exclusively as antifungal prophylaxis during conditioning and after HSCT until August 2008. As we observed a high incidence of nephrotoxicity, we changed our antifungal prophylaxis beginning from day 1 after allogeneic HSCT, from L-AmB to CAS in September 2008. Other supportive standards remained unchanged during this short period of 4.5 years.

The primary objective of this study was to evaluate safety of CAS as antifungal prophylaxis in pediatric patients following allogeneic HSCT as compared to L-AmB. The secondary objective was the incidence of aspergillosis, candidiasis, and other mycoses.

The observation period in this trial was defined as the time from start of intravenous antimycotic monoprophylaxis at the beginning of conditioning until three weeks after switching to oral antimycotic prophylaxis 3–4 days before inpatient discharge.

**Figure 1:**
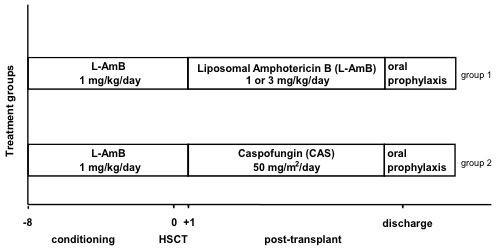
**Overview of antimycotic prophylaxis regimens.** The first group received antifungal monoprophylaxis with L-AmB, the second group with CAS, both starting on day 1 after allogeneic HSCT.

All 120 pediatric patients included in the survey received antimycotic prophylaxis with L-AmB (1 mg/kg/day) from the beginning of conditioning until day 0 (day of allogeneic HSCT) (Figure 1).

The first group of pediatric patients receiving stem cell transplants between January 2006 and August 2008 also received antimycotic prophylaxis with L-AmB after HSCT beyond that point, with an initial dose of 1 mg/kg/day. In case of fever greater than or equal to 38.5°C after the first day post HSCT, which was interpreted as the first clinical suspicion of a possible fungal infection, further antimycotic prophylaxis with L-AmB monotherapy was administered at 3 mg/kg/day, and was continued at that dosage up until oral antimycotic prophylaxis a few days before the patient’s discharge.

The second group of patients, who had undergone stem cell transplantation between September 2008 and June 2010, received the same dosage as the pediatric patients in group 1 until day 0, that is, they received L-AmB at a dosage of 1 mg/kg/day, and CAS at a dosage of 1 × 50 mg/m^2^/day intravenously beginning on day 1 after allogeneic HSCT, but not more than 50 mg/day, even in cases of fever. This dosage was based on pharmacokinetic studies, which showed that 50 mg/m^2^/day in children was comparable to 50 mg/day in adults, although a further increase to 70 mg/m²/day (maximum, 70 mg/day) would have been possible [26,27]. A loading dose of CAS was not given, since antimycotic prophylaxis had already been taking place since the start of conditioning with L-AmB at a dosage of 1 mg/kg/day. The infusion time of sixty minutes was the same for each drug. L-AmB (Ambisome®) was manufactured by Gilead Sciences (Martinsried, Germany) and CAS (CANCIDAS®) by MSD Sharp & Dohme GmbH (Haar, Germany). In addition to the systemic antifungal prophylaxis, all of the patients involved in the survey received an oral prophylaxis with amphotericin B (AmphoMoronal®) twice daily. An inhalation of antifungals did not take place.

### Study subjects

120 pediatric patients under eighteen years of age with hemato-oncological malignancies, non-malignant hematological diseases, and inborn errors of metabolism undergoing allogeneic HSCT were included in this retrospective analysis. Exclusion criteria were the presence of proven or probable invasive fungal infections before the start of conditioning according to the definitions of invasive fungal disease by the European Organization for Research and Treatment of Cancer Invasive Fungal Infections Cooperative Group and the National Institute of Allergy and Infectious Diseases Mycoses Study Group (EORTC/MSG) [28].

### Efficacy analysis

Patients were examined daily for signs of invasive fungal breakthrough infection on the ward and twice per week for three weeks after switching to oral antimycotic prophylaxis. At least once per week for the entirety of the observation period galactomannan antigens were measured by PLATELIA™ *Aspergillus* enzyme-linked immunosorbent assay (Bio-Rad Laboratories, Munich, Germany). In instances of positive galactomannan antigens, measurements were repeated again with the same blood specimen. After the confirmation of positive results, daily measurements of the galactomannan antigen took place, until a negative result was obtained.

Successful intravenous antifungal monoprophylaxis was defined as the absence of proven or probable fungal infection from the post-transplant period through the date of discharge, as well as three weeks after the end of intravenous antifungal monoprophylaxis.

### Safety analysis

This endpoint was comprised of clinical and laboratory parameters. Clinical side effects were recorded according to current Common Toxicity Criteria by the US National Cancer Institute [29]. Hepatic toxicity was analyzed by assessment of alanine aminotransferase (ALT, normal value ≤ 39 U/L) and aspartate aminotransferase (AST, normal value ≤ 39 U/L), alkaline phosphatase (AP, normal value ≤ 320 U/L), total and direct bilirubin in the patients’ sera. Renal function was assessed by plasma creatinine (normal value ≤ 0.7 mg/dl), urea (normal value ≤ 46 mg/dl) and potassium. Increases of ≥ 1.5 and ≥ 2.5 times the normal values (beginning on day 1 after allogeneic HSCT) were considered significant, or with regard to potassium, a decrease of plasma concentrations ≤ 3.4 mM or ≤ 2.4 mM. Furthermore, we appraised the need for oral substitution of potassium, bicarbonate, and calcium at discharge. We also assessed if cessation of either prophylaxis was necessary due to toxicity.

### Statistical analysis

All 120 pediatric patients who received monoprophylaxis with L-AmB or CAS were included in the assessments of safety and efficacy. The statistical comparison of the difference between the results and normal values was done with the one-sample t-test. The Wilcoxon matched pairs signed rank test was applied for a statistical comparison of these parameters: between the baseline before conditioning and day 0, the baseline and maximum/minimum during intravenous antifungal prophylaxis, the baseline and end of intravenous antifungal prophylaxis, day 0 and maximum/minimum, day 0 and end, and maximum and end. The hepatic and renal function data are presented as mean values ± standard deviation. P values of ≤ 0.05 (*), P ≤ 0.01 (**) and P ≤ 0.001 (***) were defined as statistically significant. The Two Independent Proportion Z-Test was used to compare percentage of patients with hypokalemia during intravenous antimycotic prophylaxis as well as the percentage of electrolytes substitution between two groups. Graphs were created with GraphPad Prism 4 for Windows, version 4.03 (GraphPad Software, San Diego, California, USA). The statistical analysis was carried out with the statistical program XLStat2010 (AddinSoft, Paris, France).

## Results

### Patients

In total, 120 pediatric patients under the age of eighteen years, divided into two groups, were evaluated in this analysis. All pediatric patients included in the survey, a total of 60 patients in the first group and an additional 60 patients in the second group, received antimycotic prophylaxis with L-AmB (1 mg/kg/day) from the beginning of conditioning until day 0. The 60 pediatric patients in group 1 underwent allogeneic HSCT between January 2006 and August 2008, and received L-AmB at a dosage of 1 mg/kg/day as antifungal prophylaxis from day one after allogeneic HSCT. In 34 (56.7%) of the 60 pediatric patients in the L-AmB group, the antimycotic prophylaxis with L-AmB was adjusted after HSCT to 3 mg/kg/day from the initial dosage of 1 mg/kg/day due to fever greater than or equal to 38.5°C. The second group also consisted of 60 pediatric patients, all of whom were treated intravenously with CAS at a dosage of 1 × 50 mg/m^2^/day beginning on day one after allogeneic HSCT, but no more than 50 mg/day, between September 2008 and June 2010. Fever greater than or equal to 38.5°C came about in 27 (43%) of the 60 pediatric patients treated with CAS.

The median age in the L-AmB group was 7.5 years (range 4 months to 17.6 years), while the CAS group had a median age of 9.5 years (range 8 months to 17.5 years) (Table 1). Males were slightly over-represented (L-AmB: n = 38, CAS: n = 33) in both groups. The most common primary diagnoses in both groups were acute lymphoblastic leukemia (ALL) (n = 18, n = 17) and solid tumors including Ewing sarcoma, neuroblastoma, and rhabdomyosarcoma (n = 9, n = 10). 28.3% (n = 17) of patients in the L-AmB group and 25% (n = 15) of patients in the CAS group received a myeloablative conditioning regimen with total body irradiation.

**Table 1:** Patient characteristics

**Characteristic**	**L-AmB (n = 60)**	**CAS (n = 60)**
	**No. of patients (%)**	
Gender		
male	33 (55.0)	38 (63.3)
female	27 (45.0)	22 (36.7)
Age group		
< 6 yr.	20 (33.3)	17 (28.3)
6 - 11 yr.	24 (40.0)	20 (33.3)
12 - < 18 yr.	16 (26.7)	23 (38.3)
Donor		
MUD	20 (33.3)	19 (31.7)
MMUD	4 (6.7)	1 (1.7)
MMFD	29 (48.3)	23 (38.3)
MFD	7 (11.7)	17 (28.3)
Primary diagnosis		
ALL	18 (30.0)	17 (28.3)
AML	7 (11.7)	1 (1.7)
JMML	3 (5.0)	2 (3.3)
CML	3 (5.0)	-
MDS	6 (10.0)	5 (8.3)
NHL	1 (1.7)	3 (5.0)
Solid tumors	9 (15.0)	10 (16.7)
Aplastic anemia	1 (1.7)	10 (16.7)
Neurometabolic diseases	5 (8.3)	5 (8.3)
Immunodeficiency	4 (6.7)	7 (11.7)
Chediak-Higashi-syndrome	2 (3.3)	-
Morbus Kostmann	1 (1.7)	-
Graft-versus-Host Disease		
Grade I	22 (36.7)	24 (40.0)
Grade II	4 (6.7)	5 (8.3)
Grade III	2 (3.3)	2 (3.3)
Grade IV	2 (3.3)	2 (3.3)
Chronic limited	1 (1.7)	3 (5.3)
Chronic extensive	1 (1.7)	1 (1.7)

Abbreviation*:* ALL*,* acute lymphoblastic leukemia; AML acute myelogenous leukemia; CAS, caspofungin; CML, chronic myeloid leukemia; JMML*,* juvenile myelomonocytic leukemia; L-AmB, liposomal amphotericin B; MDS*,* myelodysplastic syndromes; MFD, matched family donor; MMFD, mismatched family donor; MMUD, mismatched unrelated donor; MUD, matched unrelated donor; NHL, Non-Hodgkin lymphoma.

Leukocytopenia was observed for a median duration of 12 days in both groups. 56.7% (n = 34) of the pediatric patients in the L-AmB group and 21.7% (n = 13) in the CAS group received methylprednisolone during conditioning. 61.7% (n = 37) of patients in the CAS group were treated with prednisolone after transplantation in comparison to 38.3% (n = 23) in the L-AmB group. The median duration of intravenous antifungal monoprophylaxis was approximately the same for each group, 23.0 days (range 9–72 days) in the L-AmB group and 24.0 days (range 14–49 days) in the CAS group after allogeneic HSCT.

### Evaluation of efficacy

Prophylaxis was effective with L-AmB as well as with CAS. There was no incidence of proven invasive aspergillosis or another invasive fungal infection in either group. This also remained true during the conditioning phase, when both groups received liposomal amphothericin B, and also during the post-transplant period, in which group one received L-AmB and group two CAS. Furthermore, no proven fungal breakthrough infections were observable in either group three weeks after the conclusion of intravenous antifungal prophylaxis.

In the CAS group, one severely immunocompromised pediatric patient showed clinical and serological indicators of a probable invasive fungal infection, i. e. increase of galactomannan antigen in more than two consecutive blood samples and signs of pulmonary aspergillosis in computed tomography thirteen days after start of monoprophylaxis with CAS. However, bronchopulmonary lavage failed to detect fungi by culture or PCR. The patient died on day 94 after allogeneic HSCT of veno-occlusive disease (VOD), the parents did not consent with an obduction. There was no incidence of probable invasive fungal infections in the L-AmB group.

None of the patients in both cohorts died from an invasive fungal infection during the observation period. In addition to the single pediatric patient from the CAS group who succumbed to VOD, one patient from the L-AmB group died from a relapse of AML three weeks after the end of the intravenous antifungal prophylaxis.

### Safety and tolerability

Clinical side effects directly related to intravenous treatment with L-AmB were observed in 5 (8.3%) and directly related to CAS in 2 (3.3%) pediatric patients (Table 2). The clinical side effects in der L-AmB group occurred equally as frequently in the pediatric patients receiving L-AmB at a dosage of 1 mg/kg/day (n = 2) as in those receiving 3 mg/kg/day (n = 3).

**Table 2:** Clinical and laboratory adverse events of antifungal prophylaxis

**Variable**	**L-AmB (n = 60)**	**CAS (n = 60)**	**Difference (95% CI)**	
	**No. of patients (%)**		**Percentage points**	**p-Value**
Drug-related adverse events				
Clinical (Total)	5 (8.33)	2 (3.33)	5.0 (−3.4 to 13.4)	0.243
Fever	1 (1.67)	1 (1.67)	0.0 (−4.6 to 4.6)	1.0
Nausea	1 (1.67)	0	1.7 (−1.6 to 4.9)	0.315
Headache	2 (3.33)	1 (1.67)	1.7 (−3.9 to 7.3)	0.559
Bone pain	1 (1.67)	0	1.7 (−1.6 to 4.9)	0.315
Increase in alanine aminotransferase				
≥ 1.5 × normal value 39 U/L	13 (21.67)	14 (23.33)	−1.7 (−16.6 to 13.3)	0.827
≥ 2.5 × normal value 39 U/L	3 (5.0)	8 (13.33)	−8.3 (−18.7 to 2.0)	0.144
Increase in aspartate aminotransferase				
≥ 1.5 × normal value 39 U/L	15 (25.0)	19 (31.67)	−6.7 (−22.7 to 9.4)	0.416
≥ 2.5 × normal value 39 U/L	6 (10.0)	2 (3.33)	6.7 (−2.2 to 15.5)	0.140
Increase in alkaline phosphatase				
≥ 1.5 × normal value 320 U/L	0	0	0	1.0
≥ 2.5 × normal value 320 U/L	0	0	0	1.0
Increase in total bilirubin				
≥ 1.5 × normal value 1.1 mg/dl	3 (5.0)	0	5.0 (−0.6 to 10.6)	0.079
≥ 2.5 × normal value 1.1 mg/dl	0	0	0	1.0
Increase in direct bilirubin				
≥ 1.5 × normal value 0.3 mg/dl	9 (15.0)	5 (8.33)	6.7 (−4.8 to 18.2)	0.255
≥ 2.5 × normal value 0.3 mg/dl	3 (5.0)	2 (3.33)	1.7 (−5.5 to 8.8)	0.648
Increase in creatinine				
≥ 1.5 × normal value 0.7 mg/dl	0	0	0	1.0
≥ 2.5 × normal value 0.7 mg/dl	0	0	0	1.0
Increase in urea				
≥ 1.5 × normal value 46 mg/dl	3 (5.0)	2 (3.33)	1.7 (−5.5 to 8.8)	0.648
≥ 2.5 × normal value 46 mg/dl	0	0	0	1.0
Decrease potassium				
≤ 3.4 mmol/L	46 (76.67)	34 (56.67)	−20.0 (−3.1 to −36.9)	0.02
≤ 2.4 mmol/L	2 (3.33)	0	−3.3 (1.2 to −7.9)	0.154

Abbreviation*:* CAS, caspofungin; L-AmB, liposomal amphotericin B

**Figure 2:**
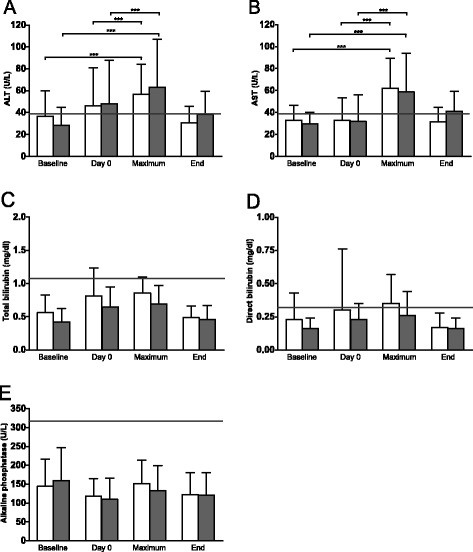
**Hepatotoxicity.** Values are shown at baseline before conditioning (baseline), day 0, maximum during therapy (maximum), and end of intravenous antimycotic monoprophylaxis (end) with L-AmB (open bars) and CAS (shaded bars). **A**: Mean and standard deviation (SD) of plasma alanine aminotransferase (ALT) (normal value ≤ 39 U/L), **B**: Plasma aspartate aminotransferase (AST) (normal value ≤ 39 U/L), **C**: Total plasma bilirubin (normal value ≤ 1.1 mg/dl), **D**: Direct plasma bilirubin (normal value ≤ 0.3 mg/dl), and **E**: Plasma alkaline phosphatase (AP) (normal value ≤ 320 U/L). The horizontal line shows the normal value. Differences were tested for significance by the Wilcoxon Matched-Pairs Signed Rank Test (Table 2).

**Figure 3:**
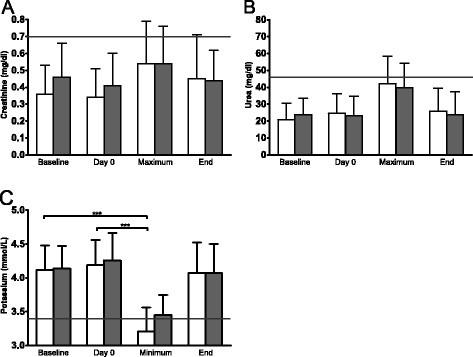
**Nephrotoxicity.** Values are shown at baseline before conditioning (baseline), day 0, maximum during therapy (maximum / minimum), and end of intravenous antimycotic monoprophylaxis (end) with L-AmB (open bars) and CAS (shaded bars). **A**: Mean and standard deviation (SD) of serum creatinine (normal value ≤ 0.7 mg/dl), **B**: Urea (normal value ≤ 46 mg/dl), **C**: Potassium (normal value > 3.4 mmol/L). The horizontal line shows the normal value. Statistical analysis by the Wilcoxon Matched-Pairs Signed Rank Test.

In the L-AmB group this included one incidence each of fever, nausea, and bone pain, as well as two cases of headache. L-AmB prophylaxis was discontinued in the latter four of these cases and switched to CAS. In the CAS group, fever occurred in one case and headache in another. We did not stop the administration of a drug in either of these two cases. Laboratory parameters of hepatic function showed a statistically significant, yet transient increase in ALT and AST in both groups, beyond the upper normal limit during treatment post-transplant in relation to baseline (P < 0.001) as well as to day 0 (P < 0.001) (Figure 2). An increase ≥ 1.5 times the normal value of 39 U/L occurred almost with the same frequency in both groups for ALT (L-AmB: n = 13, CAS: n = 14) and more frequently in the CAS group for AST (L-AmB: n = 15, CAS: n = 19) (Table 2). ALT values ≥ 2.5 times the normal value of 39 U/L were observed in 3 patients under L-AmB and in 8 patients under CAS. 6 patients in the L-AmB group and 2 patients in the CAS group showed AST levels at least 2.5fold above the normal limit. Total bilirubin and direct bilirubin also increased insignificantly during post-transplant treatment. AP did not increase during post-transplant period. In both groups, renal function parameters showed an increase in creatinine and urea during treatment with L-AmB, but not significantly beyond the upper normal limit. Hypokalemia ≤ 3.4 mmol/L occurred significantly (P = 0.006) more often in the L-AmB group (48 patients, 80%) than under CAS (34 patients, 57%) (Figure 3).

### Substitution requirement of electrolytes

A total of 25% (n = 15) of pediatric patients in the L-AmB group required oral potassium supplementation and spironolactone upon discharge. This compares to only 11.7% (n = 7) in the CAS group. Sodium bicarbonate substitution was required in 5 (8.33%) and calcium in 3 (5%) cases upon discharge in the L-AmB group. In the CAS group, calcium was given in 2 (3.3%) cases and sodium bicarbonate in one (1.7%) case. In the L-AmB group, there was a trend towards higher percentage of patients requiring potassium (P = 0.0591) and bicarbonate substitution (P = 0.094) after end of intravenous antifungal prophylaxis. All pediatric patients in the L-AmB group who required oral supplementation with potassium and bicarbonate had received L-AmB after HSCT at a dosage of 3 mg/kg/day.

## Discussion

Clinical practice guidelines have been published and adapted over the years for primary antifungal prophylaxis in adult patients undergoing allogeneic HSCT [7]. Yet primary antifungal prophylaxis in pediatric patients is based solely on recommendations resulting from small data sets reaching evidence categories of C-III or B-II [10,23,24].

In our BMT unit, children receive a comparably low dose of 1 mg/kg/day L-AmB during conditioning in order to reduce drug interactions. When using L-AmB at a therapeutic dosage (3 mg/kg/day) in pediatric patients after allogeneic HSCT, we often observed an increased need for supplementation of potassium, bicarbonate, and calcium during and within the first two weeks following intravenous antifungal prophylaxis. In a randomized, double-blind, multinational trial, CAS was better tolerated than L-AmB in 556 adults with persistent fever and neutropenia [30]. CAS in combination with other antifungal compounds, has been proven an effective option for prolonged treatment periods for invasive fungal infections in children and adolescents [31-35]. A medical record review reported the effectiveness and tolerability of CAS as primary antifungal prophylaxis in 123 severely immunosuppressed adults undergoing stem cell transplantation [36]. Therefore, we switched to CAS as primary prophylaxis after conditioning for allogeneic HSCT. An initial loading dose was not given since the pediatric patients already received antifungal prophylaxis with L-AmB during conditioning.

Although caspofungin showed good efficacy at a dosage of 50 mg/m^2^/day up to a maximum of 50 mg/day, the case of probable aspergillosis might argue to increase the dose of caspofungin to 70 mg/m²/d (maximum, 70 mg/day) in children due to pharmacological studies and its low toxicity [26,27]. The spectrum of clinical drug-related side effects in this setting included fever and headache during CAS treatment, but there was no discontinuation of CAS administration. In a prospective trial of pediatric patients (n = 49) with invasive fungal infections, fever and rash were observed during CAS treatment [22]. In our survey, five of the patients who received L-AmB experienced fever, headache, nausea, and bone pain. In four of these cases we discontinued antifungal treatment with L-AmB. A transient increase of AST and ALT occurred in our and almost all other trials when pediatric patients received CAS [22,24,34]. Creatinine and urea were not significantly altered in both groups during and at the end of prophylaxis with L-AmB or CAS. However, an elevation in serum creatinine at the end of treatment in 17.7% of treatment courses with L-AmB was observed in a single center prospective observational study of 84 pediatric patients and adults [10]. In the same study, about 50% of pediatric patients had an electrolyte wasting, defined as any hypokalemia, hypomagnesemia or both. These results were consistent with our study: the percentage of children with hypokalemia requiring oral supplementation following L-AmB treatment was significantly higher than after CAS treatment.

## Conclusions

This is the first retrospective observational trial of antifungal prophylaxis after allogeneic HSCT that has been carried out with a large cohort of pediatric patients under eighteen. In this retrospective survey, the efficacy of antifungal prophylaxis was good in both groups, since no proven invasive fungal infection occurred in either group. During the early transplant period, patients received several antibiotics and virostatics, which did not affect the tolerability of CAS. The combination of L-AmB with certain virostatics such as ganciclovir, foscarnet or cidofovir may have potentiated nephrotoxicity requiring prolonged therapeutic intervention more often here than in the CAS group. The present study is limited in that it was not randomized, and it retrospectively analyzed two periods of time, where minimal changes in daily management cannot be precluded. Prospective studies with larger cohorts due to the low incidence of invasive aspergillosis in this setting must be undertaken in order to derive clinical guidelines on antifungal prophylaxis in children undergoing stem cell transplantation.

## Competing interest

The authors declare that they have no competing interest.

## Redundant publication

No substantial overlapping with previous papers.

## Authors’ contributions

MD carried out the clinical analysis, performed the statistical analysis and drafted the manuscript. UH performed a portion of the clinical analysis. AE assisted in both the clinical and statistical analyses. PL helped to draft the manuscript. RH helped to draft the manuscript. IM conceived of the study, and participated in its design, and helped to draft the manuscript. All authors read and approved the final manuscript.

## Pre-publication history

The pre-publication history for this paper can be accessed here:

http://www.biomedcentral.com/1471-2334/12/151/prepub
